# Meander Designer: Automatically Generating Meander Channel Designs

**DOI:** 10.3390/mi9120625

**Published:** 2018-11-27

**Authors:** Andreas Grimmer, Philipp Frank, Philipp Ebner, Sebastian Häfner, Andreas Richter, Robert Wille

**Affiliations:** 1 Institute for Integrated Circuits, Johannes Kepler University Linz, 4040 Linz, Austria; p-ebner@gmx.at (P.E.); robert.wille@jku.at (R.W.); 2 Chair of Microsystems, Institute of Semiconductors and Microsystems, Technische Universität Dresden, 01062 Dresden, Germany; philipp.frank@gmx.com (P.F.); Sebastian.Haefner@mailbox.tu-dresden.de (S.H.); andreas.richter7@tu-dresden.de (A.R.)

**Keywords:** Computer-Aided Design, Microfluidic Design Automation, Meander Design, Layout

## Abstract

Microfluidics continues to bring innovation to the life sciences. It stimulates progress by enabling new ways of research in biology, chemistry, and biotechnology. However, when designing a microfluidic device, designers have to conduct many tasks by hand—resulting in labor-intensive processes. In particular, when drawing the design of the device, designers have to handle re-occurring entities. Meander channels are one example, which are frequently used in different platforms but always have to fit the respective application and design rules. This work presents an online tool which is capable of *automatically* generating user-defined, two-dimensional designs of fluidic meander channels facilitating fluidic hydrodynamic resistances. The tool implements specific design rules as it considers the user’s needs and fabrication requirements. The compliance of the meanders generated by the proposed tool is confirmed by fabricating the generated designs and comparing whether the resulting devices indeed realize the desired specification. To this end, two case studies are considered: first, the realization of dedicated fluidic resistances and, second, the realization of dedicated mixing ratios of fluids. The results demonstrate the versatility of the tool regarding application and technology. Overall, the freely accessible tool with its flexibility and simplicity renders manual drawing of meanders obsolete and, hence, allows for a faster, more straightforward design process.

## 1. Introduction

Microfluidics is an emerging field which provides technological advances to the life sciences. It is an enabling technology when it comes e.g., to biological cell studies [1,2,3], high throughput drug development [4,5,6], and diagnostic screenings [7,8,9]. Furthermore, microfluidics is advantageous over conservative methods as it uses lesser reagent and sample volumes, facilitates shorter reaction times, and provides new levels of information and detail [10,11,12,13,14].

Despite these promises, the design process of microfluidics is still in its infancy. In fact, most designs are entirely derived by hand, which requires the consideration of a substantial number of interdependencies affecting the intended behavior (Note that recently an automatic method for determining the required resistances of channels for certain applications have been proposed in [15]). This, however, leads to the situation that the design of a microfluidic device or the integration of components can currently mainly be accomplished by experts only [16] and additionally constitutes a labor-intensive process.

In order to make microfluidics more accessible (particularly for end-users, i.e., biologists, chemists, or medical experts), methods for *Computer-Aided Design* (CAD)—sometimes also called *Microfluidic Design Automation* (MDA)—are becoming essential. This can nicely be seen when looking at the microelectronics industry, which, nowadays, heavily depends on tools that allow for (automatically) modeling and simulating electrical circuits as well as to consecutively derive a physical design with fabrication details in an automated fashion.

This motivates the development of similar CAD methods and tools for the microfluidic domain, which will aid the design process, predict the designed functionality, and/or perform tasks in an automated way. Adequate tools will increase the accessibility, reduce the costs of the design process, and allow for a short time-to-market [17,18]. Corresponding methods can eventually enable microfluidics to achieve the same breakthrough as it has been observed in microelectronics.

However, currently, there is no (or not yet a) standardized design process for microfluidics. This can be explained by the rich set of available platforms which utilize different physical effects and technologies [19] and therefore all rely on their own set of design rules. These circumstances lead to a paradox situation: on one hand, microfluidics needs concepts for automation of the design process to be attractive to end users, but, on the other hand, demands the highest flexibility and full control of the design process. Particularly when developing new applications and experiments, the iteration rate for optimization is essential and quite high.

However, even though an integrated design flow aided by CAD methods does not yet exist (existing CAD solutions either address a specific problem as e.g., [20,21,22] or address a single platform as e.g., [23,24,25,26,27]), there are many re-occurring entities in each design. In particular, meander channels are a central microfluidic entity which frequently occurs in many different platforms such as pressure driven, droplet-based, and paper-based microfluidics. Meander channels and the hydraulic resistance they embody facilitate a number of functions on a microfluidic chip. More precisely, these serpentine-shaped channels are utilized to accomplish a delay of flow [28], set mixing ratios in diluter networks [29,30] or to adjust the overall operating point of a system [13]. However, even for this frequently re-occurring entity, designers still have to *manually* draw the meander channel for their respective application (e.g., ensuring a desired resistance and area constraints) and design rules (e.g., channel section, minimum bend radius, and minimum lateral channel distance). Up to now, microfluidic designers still have to use design tools such as AutoCAD, Solid Edge, or Inkscape to draw the meander design consisting of lines and radii [31]. Here, modern design tools at least allow to define parameterized models, which allow for quick adoptions of design features [32], but these models are limited to a single CAD tool and, hence, are not generally applicable. Moreover, to the best of our knowledge, no model for meanders exists yet. Consequently, no automatic tool exists that allows for generating meander designs for microfluidics.

This work presents a tool called *Meander Designer*, which automates the tedious task of drawing the fabrication design of meander channels while still retaining the full control over the design. Moreover, the tool is realized as an online tool using JavaScript running on a HTML webpage which can be accessed freely (see http://iic.jku.at/eda/research/meander_designer/). The tool allows designers to automatically generate meander channels with a *desired fluidic resistance* for their respective needs and by taking restrictions from the fabrication process into account. The Meander Designer generates the desired design of the channel and outputs this design in the widely applicable, vector-based *Scalable Vector Graphics* (SVG) file format (allowing to download the design and, afterwards, to import into design tools). This allows for integrating and connecting the meander channel into the overall design and gives designers the highest flexibility with respect to the applied design process as well as to the used fabrication technology (i.e., it is applicable for soft lithography, milling, 3D-printing, paper microfluidics, etc.).

In order to confirm the compliance of the obtained designs (i.e., to validate that fabrications of the generated designs indeed realize the desired specification), a set of meanders is generated and fabricated for the following two case studies:

- *Realizing dedicated fluidic resistances*: This case study considers the application of generating meanders with a dedicated fluidic resistance ranging from 10 to $50\;\mathrm{mbar}\;\min\;\mu L^{-1}$. To this end, the proposed tool is used to generate the designs, which are afterwards fabricated in polydimethylsiloxane (PDMS). Then, their actual fluidic resistances is systematically measured and compared to the desired values.
- *Realizing dedicated mixing ratios of fluids*: This case study considers the application of generating fluidic mixtures of different ratios. Therefore, a design is used containing two generated meanders with a dedicated resistance ratio, which eventually results in the desired mixing ratio. The mixing ratios 20:20, 40:20, 30:10, and 40:10 are tested, which also represent the used fluidic resistances in $\mathrm{mbar}\;\min\;\mu L^{-1}$ of the two meanders. The resulting designs are fabricated and, afterwards, the mixing ratios are measured.

The obtained measurements taken from the fabricated devices confirm that the meanders automatically generated by the Meander Designer indeed yield designs that can directly be used as entities in the overall design.

The remainder of this paper is structured as follows: Section 2.1 first introduces the Meander Designer including a detailed problem description and technical details of the implementation. Afterwards, Section 2.2 describes the used fabrication process, which is applied to fabricate devices and, by this, confirm the compliance of the proposed tool. Details on the setup of the two case studies are provided in Section 2.3. Section 3 presents the results of the two case studies and the respectively conducted measurements. Finally, Section 4 provides a conclusion.

## 2. Proposed Tool, Fabrication Process and Setup of Case Studies

### 2.1. Meander Designer

This section describes the Meander Designer tool. Therefore, this section first states the problem description and afterwards provides technical details of the online tool.

#### 2.1.1. Problem Description

As reviewed in the introduction, meanders are central entities in microfluidic designs because they can be used to delay the flow [28], realize mixing ratios in diluter networks [29,30], or to adjust the overall operating point of a system [13]. Up to now, meanders are usually designed manually by a designer using a design tool like AutoCAD, Solid Edge, or Inkscape. However, meanders always have to fit the realized application and, hence, a large variety of different meanders is often needed.

In particular, the fluidic resistance of the meander is important as it impacts the pressure-flow relation. The fluidic resistance *R* for a meander channel with a rectangular cross section depends on its length *L*, width *W*, height *H*, and the viscosity μ of the fluid passing. The resistance for a meander with a low aspect ratio (H/W<1) is defined by [33]

(1) $$R=\frac{\alpha \;\mu \;L}{W\;H^{3}},$$

where α denotes a dimensionless parameter defined as

(2) $$\alpha =12{\left[1-\frac{192\;H}{\pi^{5}\;W}\tan h\left(\frac{\pi \;W}{2\;H}\right)\right]}^{-1}.$$

Besides embodying a particular resistance value, a meander needs to comply with specific design rules and ideally make full use of a given chip space while providing needed connectivity to the rest of the design.

Designing a meander with a desired fluidic resistance and additionally considering all those constraints makes the design a cumbersome task. For example, already a slight change in the length of the meander significantly affects the desired resistance (cf. Equation (1)).

In an effort to automate this task (and, by this, to aid the designer), a tool called *Meander Designer* is developed, which allows for automatically generating meander designs for the designer’s specific needs and constraints. Therefore, the designer only has to provide

- the desired resistance,
- the viscosity of the used fluid,
- the desired width/height ratio of the meander boundary,
- the channel width and height (information of the channel cross section),
- the fabrication constraints such as a lateral channel distance and a minimum bend radius,
- the inlet and outlet positions, as well as
- an optional correction factor in the form of a constant or first order function.

Using this input, the tool generates a meander design in a fully-automated fashion. This includes

- the meander design as a Scalable Vector Graphics (SVG) file (which is supported by all commonly used design tools),
- the resulting channel length,
- the resulting channel volume,
- the resulting boundary size of the meander (the width and height), as well as
- the logging file, which serves as a documentation of the generated meander.

Furthermore, the Meander Designer allows to account for actual and non-ideal fabrication results in the form of a correction factor. For example, in the process of soft lithography, the fabrication result e.g., of the channel width depends on a variety of parameters. Main influences are the photomask, the exposure step, the development step, and various tempering steps. The channel height also underlies variations due to coating, tempering, and development steps. Therefore, the fabrication result can vary and depends on a series of influences. This complexity of dependent and independent influences makes it difficult to account for in the design process.

Therefore, an optional correction factor is introduced to the Meander Designer. The correction factor enables the designer to account for the deviation of the in-house fabrication process that is at hand. More precisely, the designer is given the possibility to specify the correction factor in the form of a constant or as a first order function. Then, a meander which should implement the desired resistance has to be fabricated with the corrected resistance value in order to compensate the deviation. The corrected resistance is calculated as follows when a first order function is used:

(3) $$CorrectedResistance=C_{0}+C_{1}\cdot DesiredResistance.$$

Equation (3) represents a lump model of the influences originating from the fabrication process. A correction factor for the fabrication process used in this work is exemplarily described in Section 3.1.

#### 2.1.2. Description of the Online Tool

The Meander Designer is developed as an online tool which makes it broadly accessible for microfluidic designers and allows them to generate meanders without requiring any local installations. The tool is implemented in JavaScript in combination with the Bootstrap library and embedded within an HTML webpage. Users can access the tool through the link http://iic.jku.at/eda/research/meander_designer/. In the following, the input and output masks are described. Moreover, a brief overview of the tool’s internals is provided.

**Figure 1:**
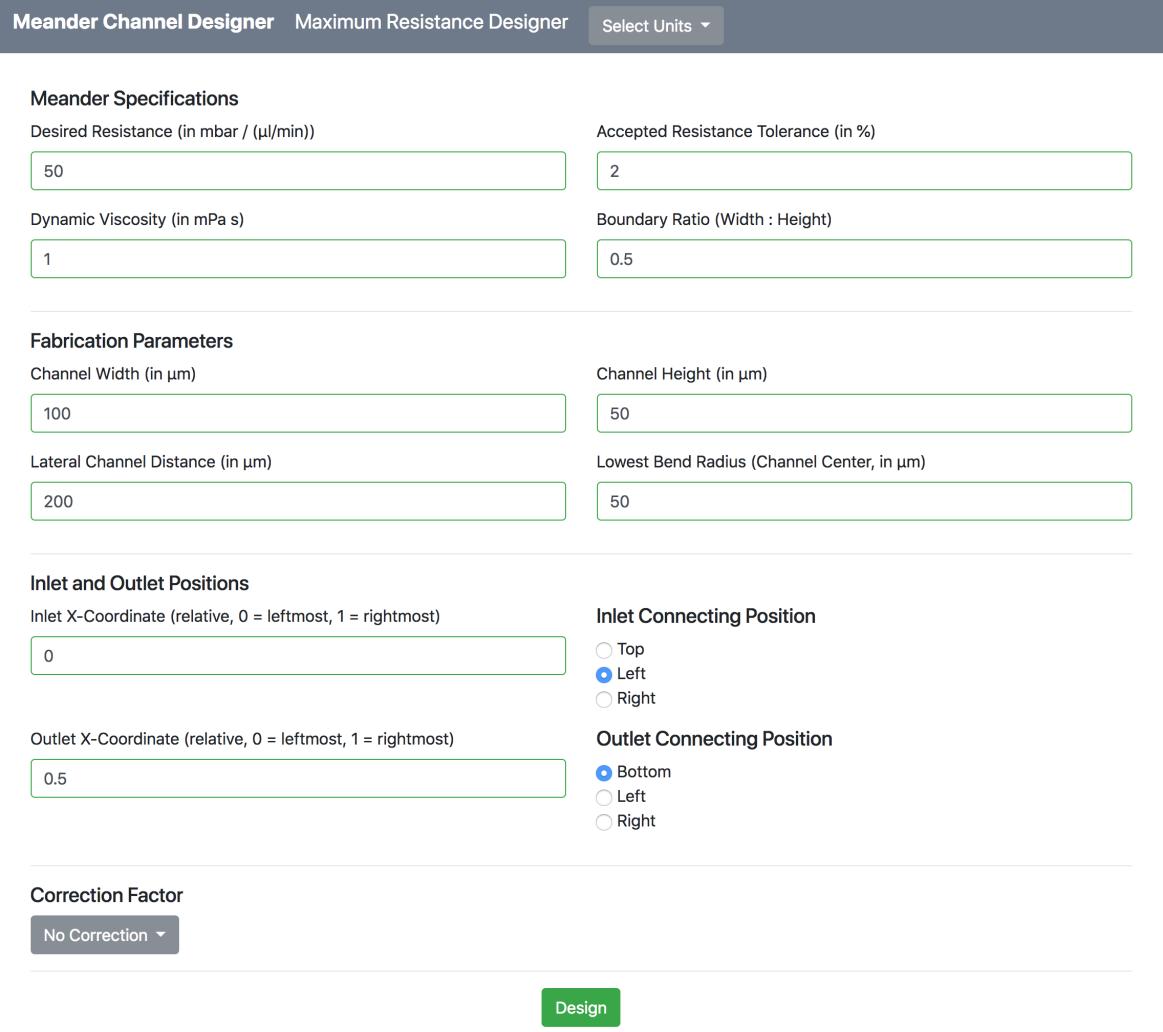
Input mask.

Figure 1 shows the input mask, which allows the designer to input the desired meander specifications, the fabrication parameters, the inlet and outlet positions, as well as the correction factor.

By pressing the green button “Design” the tool starts the design process. Therefore, the provided input parameters and Equation (1) are used in order to generate a meander with the specified resistance and constraints. Internally, the tool uses an A* search algorithm [34] and the meander is described as a closed contour. This closed contour is specified as a SVG-path, i.e., a list of coordinates which are connected by lines and curves.

**Figure 2:**
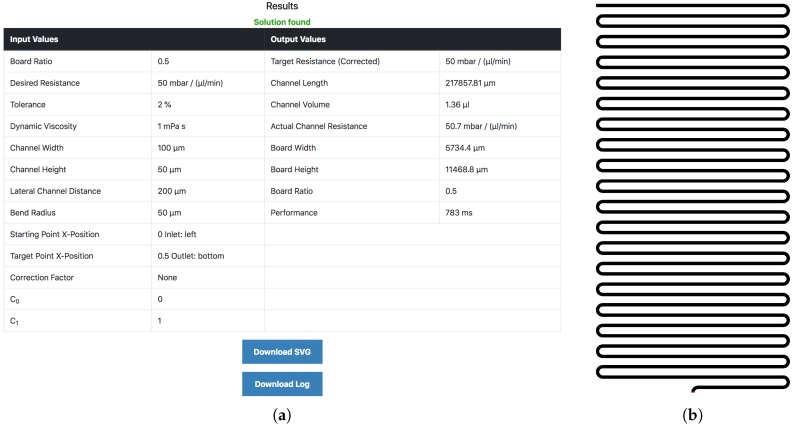
Output mask. (**a**) output log; (**b**) generated design.

Figure 2 shows the corresponding output mask. More precisely, Figure 2a shows an output log containing a documentation of the generated meander and additionally contains two download buttons allowing for obtaining the generated design as a SVG file as well as a log file (which enables reproduction of the results). Additionally, the output provides a preview of the generated meander directly in the browser, which is shown in Figure 2b. This preview allows a first assessment of the generated design.

Overall, the Meander Designer allows for automating the tedious task of manually designing a meander and is suited to seamlessly fit into any design step regardless which technological process is utilized. After generating a meander design, the obtained SVG file can be imported into the design tool. In the design tool, the generated meanders can easily be integrated and connected to the rest of the design.

### 2.2. Fabrication Process

In order to confirm the compliance of the proposed tool, case studies are conducted for which the obtained designs are fabricated and measured. The experimentally determined results are compared to the initial specification in the tool. This section briefly describes the applied fabrication process (soft lithography with master fabrication and chip fabrication).

#### 2.2.1. Master Fabrication

A glass substrate (Borofloat 33, Schott AG, Mainz, Germany) with a thickness of 1.0 mm is cleaned with acetone, isopropanol, and deionized water. The substrate is consecutively dried in a nitrogen stream. A dehydration bake at $200{\;}^{\circ }C$ for 20 min is performed using a hot plate as all following baking steps. A single layer of dry film resist (Ordyl SY355, Elga Europe, Nerviano, Italy) is laminated onto the substrate followed by a soft-bake at $85{\;}^{\circ }C$ for 3 min. The lamination process and the soft bake are repeated for another layer. The substrate is exposed with UV light through a polymer film mask (dark field) for 90 s. The post-exposure bake (PEB) follows at $85{\;}^{\circ }C$ for 60 min. The development is performed using an Ordyl developer and rinser (Ordyl Developer & Rinser, Elga Europe, Nerviano, Italy). The substrate is developed first in the used developer for 4 min and then in the fresh developer for 3 min. The development is discontinued with the rinser bath for 2 min. The development is finished by rinsing the substrate with isopropanol and deionized water. The process is completed with a hard bake at $120{\;}^{\circ }C$ for 1.5 h.

#### 2.2.2. Chip Fabrication

For the experiment, 33.0 g of PDMS (Sylgard 184, Dow Corning, Midland, MI, USA) in a ratio of 10:1 is prepared and thoroughly mixed with an electric stirrer for 5 min. The compound is degassed in a vacuum chamber for 45 min. The PDMS is poured onto the previously fabricated master mold creating a layer thickness of 4 mm. The PDMS is cured in a convection oven at $60{\;}^{\circ }C$ for 2.0 h. The chip is peeled off the master mold and bonded via oxygen-plasma (at 50 W for 2 min) onto a glass substrate.

### 2.3. Setup of Case Studies

This section describes the setup of the two conducted case studies that utilize the Meander Designer covered in Section 2.1 to (automatically) generate the desired designs and the fabrication process reviewed in Section 2.2 to realize the respective devices.

#### 2.3.1. Setup for Realizing Dedicated Resistances

The first case study considers the realization of dedicated fluidic resistances. Therefore, the Meander Designer is used to generate designs of meanders which have resistances that range between 10 to $50\;\mathrm{mbar}\;\min\;\mu L^{-1}$, i.e., 10, 15, 20, 25, 30, 40, and 50 $\mathrm{mbar}\;\min\;\mu L^{-1}$. As an example, for generating a meander with resistance of $50\;\mathrm{mbar}\;\min\;\mu L^{-1}$, the specification and fabrication parameters as shown in Figure 1 is set in the Meander Designer. The correspondingly generated output consisting of the log and the generated meander is shown in Figure 2.

Note that, when generating these meanders using the Meander Designer, the variations resulting from the used fabrication process and how they affect the resistances of the meanders were unknown. Therefore, no correction factor in the Meander Designer was initially used.

For each fluidic resistance, four copies of the generated meander are fabricated. Then, the volumetric flow rate through each of these meanders is measured. Therefore, a pump applies increasing pressures ranging from 0 mbar to 1000 mbar using a step width of 50 mbar. In order to also consider hysterical effects, the pressure is decreased with the same step width. This procedure is repeated for all four meander copies. By taking the quotient between the pressure and the volumetric flow rate, the fluidic resistances of the meanders are determined. Furthermore, the measurement setup eliminates errors in the periphery supply and the measurement equipment. Details on this measurement setup are described in Appendix A. The obtained results are discussed in Section 3.1.

#### 2.3.2. Setup for Realizing Dedicated Mixing Ratios

The second case study considers the realization of dedicated mixing ratios. Therefore, the Meander Designer is applied for generating for each design realizing a certain mixing ratio two meanders. These two meander designs realize a fluidic resistance ratio, which eventually implements a certain mixing ratio. For example, to realize a mixing ratio of 1:1 of two fluids having the same properties, the resistances of both meanders have to be equal (e.g., 20 $\mathrm{mbar}\;\min\;\mu L^{-1}$ for both meanders). In contrast, to realize a mixing ratio of 2:1, the resistances of the two meanders have to relate accordingly (e.g., 40 $\mathrm{mbar}\;\min\;\mu L^{-1}$ for one meander and 20 $\mathrm{mbar}\;\min\;\mu L^{-1}$ for the other). This case study realizes mixing ratios of 20:20, 40:20, 30:10, and 40:10, which also represent the used fluidic resistances in $\mathrm{mbar}\;\min\;\mu L^{-1}$ of the two meanders.

Therefore, dyed water with two different colors (i.e., water colored with blue and red ink) is used. A pressure pump drives both fluids and the resistance of the meander determines the amount of flow through the respective meander. For measuring the mixing ratio, pictures are taken where the width of the red colored stream and the width of the blue colored stream are measured pixel-wise with an image processing software. The mixing ratio is determined by the ratio of two pixel values. Details on this measurement setup are described in Appendix B. The obtained results are discussed in Section 3.2.

## 3. Results

This section presents and discusses the results which are obtained in the two case studies.

### 3.1. Results for Dedicated Resistances

**Figure 3:**
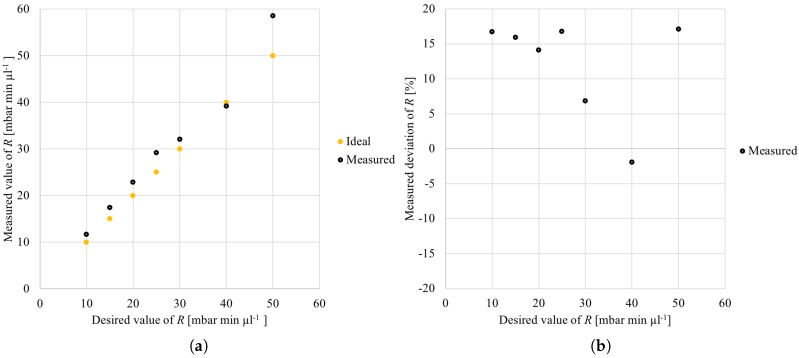
Measured resistances when the meanders are generated without correction. (**a**) absolute values of *R*; (**b**) deviation of *R*.

For the evaluation of the first case study, the generation (and fabrication) of meanders is considered, which realize different fluidic resistances ranging from 10 to $50\;\mathrm{mbar}\;\min\;\mu L^{-1}$ (in total, seven different designs are generated and fabricated). For these generated meander designs, *no* correction factor has been applied in the Meander Designer because the correction factor was unknown for the fabrication process at hand. Figure 3 shows the obtained results. More precisely, Figure 3a shows the desired resistance on the *x*-axis (i.e., the values specified in the Meander Designer), while the *y*-axis provides the actually obtained (i.e., measured) values of the resulting (fabricated) design. All obtained results are denoted by black points, while the ideal match between the measured and the desired resistances are additionally added in terms of orange points which serve as reference.

Overall, it can be observed that the measured resistance values match well with the desired resistances (although no correction factor has been used yet). However, still, it can be observed that, for all desired resistance values except for $40\;\mathrm{mbar}\;\min\;\mu L^{-1}$, the measured resistances are greater than the desired (i.e., the ideal) resistances. This result in deviations of the measured resistances compared to the desired resistances as shown in Figure 3b (the deviations are provided on the *y*-axis). The deviation is defined by the ratio between the measured and the desired meander resistance (i.e., Measured value of R/Desired value of R ·100%−100%). Therefore, the smaller the absolute value of the deviation, the better is the match between the measured and the desired resistance of the meander.

The rest of this case study aims to demonstrate how the correction factor allows for decreasing the deviation between the measured and the desired resistance value of the meander. Therefore, first the obtained measurements are used to derive a correction factor for the applied fabrication process (see also the corresponding discussion in Section 2.1.1). Here, a straight line is calculated describing the correction for the measured resistance values as a function of the desired resistance values. Therefore, the least squares method [35] is used to calculate a straight line that best corrects the measured resistance values. For the used fabrication process, this yields $C_{0}=-0.56$ and $C_{1}=0.92$, i.e., resulting in

(4) CorrectedResistance=−0.56+0.92·DesiredResistance.

In order to realize the desired resistance, the Meander Designer has to generate a design with resistance CorrectedResistance. This corrected resistance value eventually compensates the deviation resulting from the fabrication process.

**Figure 4:**
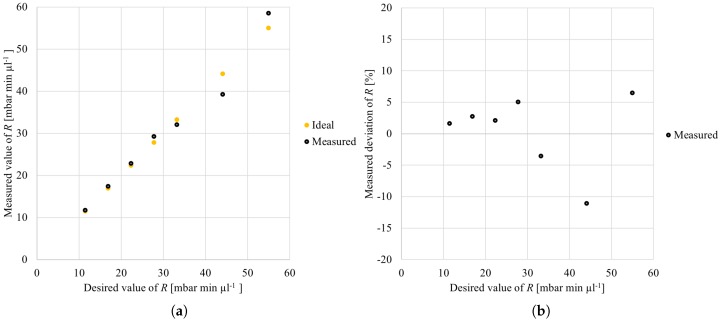
Measured resistances when the meanders are generated with correction. (**a**) absolute values of *R*; (**b**) deviation of *R*.

In this case study, the whole fabrication process and measurements of new corrected meander designs have not been repeated. Instead, this case study demonstrates the effect of the correction factor by correcting the *desired* resistances according to the correction factor. More precisely:

- Let’s assume the CorrectedResistance is equal to 10, 15, 20, 25, 30, 40, or 50.
- This allows for determining the DesiredResistance by using the lump model, which gives 11.48, 16.91, 22.35, 27.78, 33.22, 44.09, and 54.96.
- When the Meander Designer would now be applied again to realize meanders with the DesiredResistance and additionally taking the correction factor into account, exactly the designs would result as before when no correction factor was used.
- This allows for comparing the corrected desired resistances with the previously measured data set. The corresponding results are presented in Figure 4, where Figure 4a shows the absolute values and Figure 4b shows the deviations.

This correction results in an even better match between the measured and the desired resistance values. Figure 4a shows that many markers almost perfectly cover the ideal values. This also results in a reduction in the deviation of the desired resistance, which is shown in Figure 4b. In fact, after taking the correction factor into account, an overall maximal deviation of only −11.1% is obtained.

Please note that the deviations between the actual and the desired resistance values have nothing to do with the Meander Designer, but result from the used fabrication process. The Meander Designer only provides a means to correct this deviation.

Overall, the designs automatically generated by the Meander Designer provide a good basis for the fabrication process and indeed lead to fabricated designs that almost perfectly match the desired specifications.

### 3.2. Results for Dedicated Mixing Ratios

**Figure 5:**
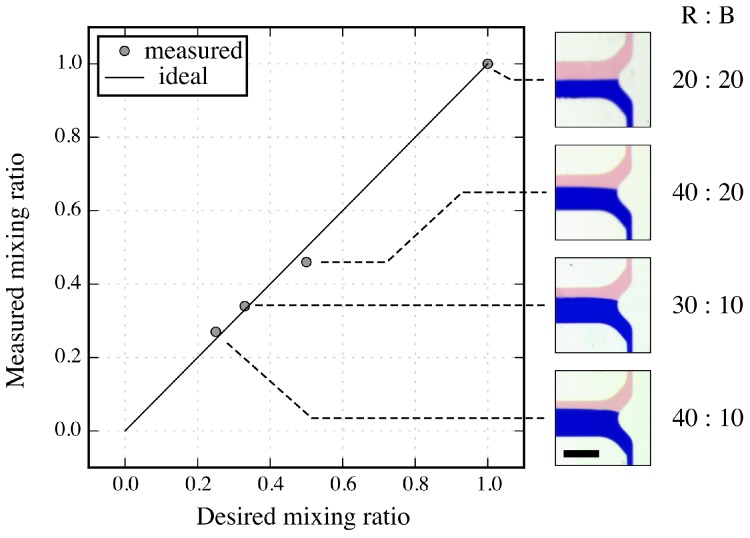
Measured mixing ratios using meanders with resistance ratios of 20:20, 40:20, 30:10, and 40:10.

For the evaluation of the second case study, the mixing ratios of the two differently dyed fluids are measured. Recall that these mixing ratios are produced by resistance ratios of meander channels. Figure 5 shows the results of the experiments measuring the mixing ratios. This plot shows on the *y*-axis the actually measured mixing ratio and on the *x*-axis the desired mixing ratio. The right-hand side additionally provides pictures of the resulting mixtures. Analyzing the widths of the blue and red colored fluids allows for determining the actually obtained mixing ratio. As can be seen in this plot, the mixing ratios are almost perfectly in line with the desired (i.e., ideal) mixing ratios. The obtained coefficient of determination is equal to $R^{2}=0.99$. These precise results allow for inferring that the deviations caused by the fabrication process are almost equalized due to the resistance ratios of the meanders (i.e., no correction factor is needed in this case).

Overall, both conducted case studies and the obtained measurements taken from the fabricated devices confirm that the designs automatically generated by the Meander Designer precisely implement the input specification and, hence, can directly be used in designs.

## 4. Conclusions

This work proposed a tool for automatically generating meander designs. This tool renders the manual drawing of meanders obsolete and, hence, allows to speed up the design of microfluidic devices. By providing the Meander Designer as an online tool (accessible through the link http://iic.jku.at/eda/research/meander_designer/) and generating the meander channels in the open SVG file format, the tool can be used by a large group of users from the microfluidic domain. The conducted case studies and the obtained results proved that the tool is capable to produce absolute fluidic resistance values as well as mixing ratios using relative fluidic resistance ratios. The obtained meanders can be utilized to generate precise fluidic devices, internal mixing ratios or to set the ideal operation point of a fluidic network. The flexibility of the tool to adjust it to any given technological process renders it very employable in any design process. Overall, the tool provides an important step towards the automation of the design process for microfluidic devices.

## Figures and Tables

**Figure A1:**
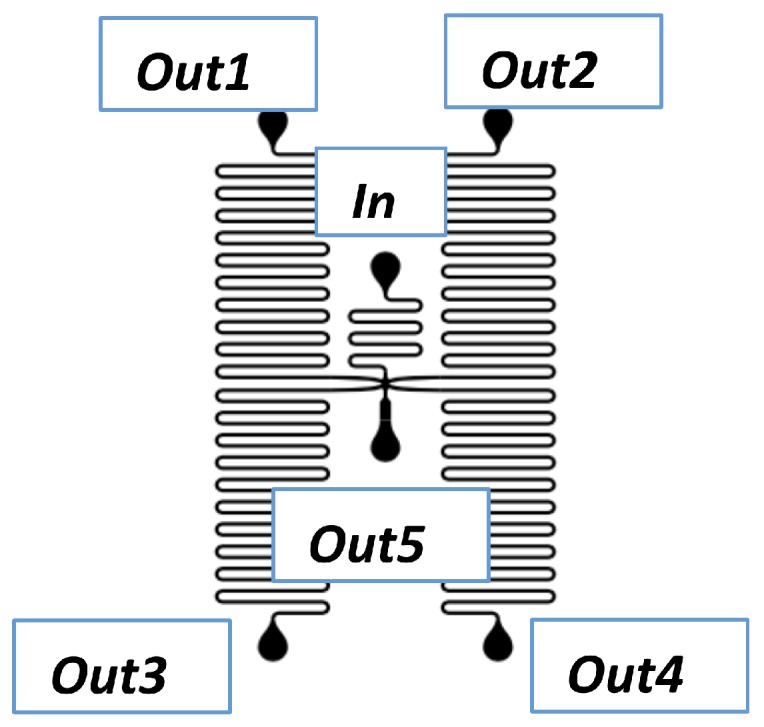
Overall design with four meanders, an input channel and dedicated inputs and outputs.

**Figure A2:**
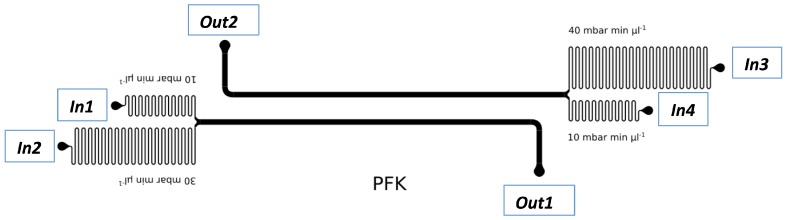
Overall design for generating mixing ratios of 30:10 and 40:10.

## Appendix A. Setup for Realizing Dedicated Resistances

The resistances of the used meander channels range from 10 to $50\;\mathrm{mbar}\;\min\;\mu L^{-1}$. Each of these generated meander designs is integrated into an overall design, which allows for measuring the fluidic resistance. Figure A1 shows the overall design for one resistance value, which consists of four (mirrored and rotated) copies of the generated meander. For the integration and connection of the four meander copies, Inkscape was used. More precisely, in the overall design, the four inputs of the meanders are connected to a common interconnection point. This common interconnection point is connected by an input channel with the input *In*. The outputs *Out1*, *Out2*, *Out3* and *Out4* are used for measuring the resistances of the four meander copies, while output *Out5* only serves for measuring the fluidic resistance of the input channel and the periphery.

Now, in order to measure the realized resistances, a fluidic pressure pump is connected to the input *In*. Furthermore, the outputs *Out1*, *Out2*, *Out3*, and *Out4* are connected by a 2-way-fluidic valve and output *Out5* is closed. The respective outputs of the four fluidic valves are merged and eventually connected to a volumetric flow sensor. For the actual measurement, a pressure is applied and only a single fluidic valve is opened, which causes the flow through a single meander and, hence, allows for measuring the fluidic resistance of a single meander.

Using this setup yields a measurement of the fluidic resistance $M_{i}$ (Equation (A1)), which additionally to the fluidic resistance *R* of the meander also contains the input fluidic resistance $R_{V}$, the fluidic resistances of the tubing $R_{tub}$, the fluidic resistance of the valve $R_{valve}$, and the fluidic resistance of the flow sensor $R_{sens}$, i.e.,

(A1) $$M_{i}=R+R_{V}+R_{tub}+R_{valve}+R_{sens}.$$

In order to extract the fluidic resistance *R*, additionally, the resistance $M_{ii}$ (Equation (A2)) of the input channel and periphery is measured. Therefore, the outputs *Out1*, *Out2*, *Out3*, and *Out4* are closed. Afterwards, the fluidic valves including the flow sensor are connected one after another to the output *Out5*. Eventually, this allows for measuring the overall resistance of $R_{V}$, $R_{tub}$, $R_{valve}$, and $R_{sens}$, i.e.,

(A2) $$M_{ii}=R_{V}+R_{tub}+R_{valve}+R_{sens}.$$

Having $M_{i}$ and $M_{ii}$ allows for obtaining the resistance of the meander (Equation (A3)), i.e.,

(A3) $$R=M_{i}-M_{ii}.$$

For measuring the resistances of the meander channels, water having a viscosity of 1 mPa s is used. This water is driven by a pressure pump, which is connected at input *In*. The pump applies increasing pressures ranging from 0 mbar to 1000 mbar using a step width of 50 mbar. Similarly, the pressure is decreased with the same step width. Each pressure value is fixed for 30 s and approximately 20 measurements are conducted in 1 s. This procedure is repeated for all four meanders contained on the microfluidic device (i.e., only a single valve is open at a time). Overall, this results for each of the considered fluidic resistances in a data set containing the current time, the set pressure, the internal pressure, the resulting volumetric flow rate, and the respective state of the fluidic valves.

For analyzing the obtained data set, a Python script is used. This script splits the data into blocks where a block contains the measurements for one dedicated pressure value (either in the pressure increasing or decreasing phase) and one meander. Using such a data block, the script determines the fluidic resistance by the quotient between the pressure and the volumetric flow rate. Here, the median value of the pressure/flow rate is used because this prevents the value from being skewed by eventual spikes in the measurements.

The results obtained by the different data blocks are afterwards further aggregated: first, the median of the fluidic resistances is built separately for the increasing and decreasing phase of the pressure for each meander. Overall, this gives eight resistance values for the two phases and the four meanders on the considered microfluidic device. Finally, again, the median is built over these eight values.

## Appendix B. Setup for Realizing Dedicated Mixing Ratios

For generating dedicated mixing ratios, two meanders are incorporated into an overall design. Figure A2 shows the design for two mixing structures allowing for generating a ratio of 40:10 and 30:10. Here, e.g., the left structure connects the two meanders to the inputs *In1* and *In2* and merges the outputs of both meanders into a wide channel. This channel is eventually used to observe the mixing ratio and ends in the output *Out1*. This is similar for the structure on the right-hand side of Figure A2.

For measuring the mixing ratios, dyed water with two different colors is used, i.e., water colored with blue and red ink. A pressure pump drives both fluids, which enter the device via *In1* and *In2*. The resistance of the meander determines the amount of flow through this respective meander.

For observing the mixing ratio, pictures are taken using a microscope and a CCD camera. The width of the red colored stream and the width of the blue colored stream in the channel where the two meanders merge are measured pixel-wise with the image processing software ImageJ. The ratio of two pixel values determines the mixing ratio.
